# BRCA1/2 mutations and outcomes among Middle Eastern patients with early-onset breast cancer in Oman

**DOI:** 10.1093/oncolo/oyae214

**Published:** 2024-08-26

**Authors:** Waleed S Al Amri, Ahmed H Al Amri, Aisha Al Abri, Thomas A Hughes, Fatma Al Lawati

**Affiliations:** Department of Histopathology and Cytopathology, Royal Hospital, P.C. 111, Muscat, Oman; National Genetic Centre, Royal Hospital, P.C. 111, Muscat, Oman; Department of Histopathology and Cytopathology, Royal Hospital, P.C. 111, Muscat, Oman; School of Science, Technology and Health, York St. John University, York YO31 7EX, United Kingdom; School of Medicine, University of Leeds, Leeds LS2 9JT, United Kingdom; Department of Histopathology and Cytopathology, Royal Hospital, P.C. 111, Muscat, Oman

**Keywords:** early-onset breast cancer, breast cancer in Oman, BRCA1/2 genes, BRCA mutations variants

## Abstract

**Background:**

High prevalence of early-onset breast cancer (EOBC) has been reported in Middle Eastern populations. For example, in Oman more than 50% of patients with breast cancer (BC) are under age 45 at diagnosis. Causes for this high incidence are unknown. Germline BRCA gene mutations have been associated with EOBC, however, prevalence of these mutations and how they relate to EOBC in Oman has not been assessed.

**Patients and Methods:**

Clinical data were collected for patients with BC treated at Royal Hospital, Oman between 2010 and 2022. Germline BRCA1/2 gene mutations were identified using sequencing and MLPA. Correlation and Kaplan-Meier survival analyses were performed to test relationships among clinico-pathological features, gene mutations, and outcomes.

**Results:**

Total of 1336 Middle Eastern patients with BC were included; 611 were aged <45 at diagnosis (45.7%). No significant correlation was found between BRCA1/2 mutation status and EOBC (*P* = .229), and the majority of EOBC cases had no family history of BC. EOBC tumors did, however, differ in clinicopathological features; EOBCs were significantly larger (*P* < .0001), of higher grade (*P* < .0001), and included more HER2-enriched, and triple negative subtypes (*P* = .018) compared with later onset cases. Accordingly, survival analyses revealed that EOBC had significantly worse disease-free survival (*P* = .002). BRCA gene variants showed a distinct range of mutations including, in BRCA2, 3 previously unreported mutations and 4 potential founder recurrent mutations.

**Conclusion:**

Our findings showed that germline BRCA1/2 mutations were not over-represented in EOBC cases in Oman, and therefore are unlikely to be responsible for high EOBC rates.

## Teaser

This is one of the largest study from the Middle Eastern region to investigate genetic causation factors for the high prevalence of early-onset breast cancer (EOBC). Findings show germline BRCA1/2 mutations are not over-represented in EOBC cases, and therefore are unlikely to be responsible for high EOBC rates.

**Implications for practice**

This is one of the largest study from the Middle Eastern region to investigate genetic causation factors for the high prevalence of early-onset breast cancer (EOBC). We showed germline BRCA1/2 mutations were not over-represented in EOBC cases, and therefore are unlikely to be responsible for high EOBC rates. Most EOBC cases had no family history of breast cancer (BC), suggesting that inherited genetics may not be the sole driver. The findings of the study suggest to expand testing of young patients with BC in the region for other hereditary BC genes. Also, to consider sequencing tumor cells for somatic mutations in these genes that are associated with EOBC.

## Background

Breast cancer is the most common malignancy in women, with more than 2 million new cases diagnosed globally and an estimated 685 000 deaths from the disease in 2020.^1^ In Oman, according to latest Oman National Cancer Registry Report, breast cancer was the most frequent cancer over the period 1996-2015, accounting for 10.9% of all cancers registered among females and males.^2^ The registry data also demonstrate that 53.5% of these breast cancers were diagnosed in patients under the age of 45,^2^ representing a very high proportion of early onset (EOBC) cases. For comparison with Western countries, EOBC cases accounted for approximately 10% of all new female breast cancer cases in US,^3^ 4% in UK,^4^ and 6% in Canada.^5^ By contrast, data from many population-based cancer registries worldwide indicate relatively high frequencies of EOBC in low/middle-income countries, such as Middle Eastern and Asian countries, as compared to high-income countries. The observed median age for female breast cancer diagnoses in low/middle-income countries has been reported as a decade earlier than in high-income countries,^6^ and rates of EOBC have been estimated at up to 20%.^7^ Even in this context, the reported incidence of EOBC in Oman remains extraordinarily high. It should be noted that comparison of EOBC rates in the wider literature can be confused by use of different cutoff ages (40, 45, or 50 are all used), although the US Centre for Disease Control and Prevention has suggested a standardized definition of younger than 45; we have followed this definition.

Various factors may play roles in defining the high rates of EOBC in Middle Eastern countries. Younger people can make up a relatively high proportion of the overall population, as compared to many high-income Western countries, and this can lead to raised EOBC rates.^8^ Psychosocial and cultural factors may also contribute to under-reporting of the incidences^9^ particularly in older patient groups,^10,11^ thereby potentially rising the reported EOBC proportion. However, other factors are needed to explain the high prevalence of EOBC in Oman specifically, and in the wider region, potentially including family history, lifestyle, and genetic factors.

Some genetic factors are known to increase lifetime risk of BC, and disproportionately raise the risk of developing EOBC. The BRCA1 and 2 genes are the best-known examples; these genes harbor a wide-range of germline pathogenic mutations that give a very strong penetrance for occurrence of BC overall (> 50% depending on population) with the majority of that risk within early onset years.^12,13^ However, even for these very well-understood genes, there is a lack of data concerning their relevance in Middle East and North Africa (MENA) populations. Furthermore, an increasing list of other genes have been shown to have rarer germ-line variants that are associated with strongly increased EOBC risk; these include ATM, PALB2, CHEK2, PTEN, and TP53.^14^ Some centers now test for these variants in individuals with otherwise unexplained strong family histories of BC, and may offer increased and earlier monitoring for BC if variants are identified.^15^

Detailed clinicopathological, survival, and genetic characterization of large cohorts of patients with BC in the MENA region are lacking, especially with respect to the high incidence of EOBC. In this study, we present one of the largest studies from the region, conducted in Oman, to characterize patients with BC at clinico-pathological and genetic levels. To the best of our knowledge, this is the first study attempting to investigate causation factors for high prevalence of EOBC in an Arab country, by investigating whether BRCA1/2 gene mutations are associated with EOBC cases. In addition, we stratified our cohorts with clinco-pathological parameters, survival outcomes, and the BRCA mutational spectrum.

## Materials and methods

### Study population and clinical data

Patients diagnosed with BC at the National Oncology Centre (NOC) at the Royal Hospital, Oman from 2010 and 2022 were identified from their electronic health records (EHR) and data concerning their personal, family history, clinical and histopathological information were extracted (*n* = 1336). The study inclusion criteria were as follows; treated within the NOC, Arab ancestry from the Gulf region of the Middle East, and data available within the EHR at the Royal Hospital. Genetic testing for BRCA gene mutations has been ongoing since 2014 at the National Genetic Centre, Royal Hospital, Oman. Patients with BC attending the NOC were from all regions in the country. They were offered genetic testing and patients with high risk for familial breast/ovarian cancer according to National Institute for Health and Care Excellence (NICE) UK guidelines for BRCA gene testing were encouraged to participate. Genetic counseling was offered when mutations were identified. Disease-free survival (DFS) and overall survival (OS) were used to measure the survival rates from date of diagnosis confirmed by histopathological report until date of recurrence or death, respectively. Family history of BC was regarded as positive if patients reported first- or second-degree relatives who had BC.

### DNA isolation

Blood samples were collected using EDTA vacutainer tubes. DNA isolation was carried out using a magnetic bead-based method using the Chemagic Star analyzer (Hamilton), following the manufacturer’s protocol. DNA quantitation was carried out using the Qubit dsDNA BR Assay (Thermo Fisher Scientific).

### Targeted capture sequencing of BRCA1/2 genes

Genomic target BRCA1/2 regions were amplified using the Ion AmpliSeq Library Kit and Ion AmpliSeq BRCA1/2 Community panel (Thermo Fisher Scientific). This utilizes 167 amplicons for analysis of the coding regions of both BRCA1 and BRCA2 genes. The resulting amplicon DNA was treated with the FuPa reagent (Thermo Fisher Scientific), to partially digest the primers and phosphorylate the amplicons. Amplicons were then ligated to ion adapter barcodes, and purified. Libraries were quantified by qPCR without further amplification. A purification step was carried out using the AgencourtAMPure XP PCR purification system (Beckman Coulter). AgencourtAMPure XP utilizes an optimized buffer to selectively bind DNA fragments of 100bp and larger to paramagnetic beads. Excess primers, nucleotides, salts, and enzymes were removed by washing. Sequencing was carried out on the ION S5 analyzer (Thermo Fisher Scientific).

### PCR and Sanger sequencing

Primers were designed using Primer3 (https://primer3.ut.ee/) to amplify the regions of interest of the BRCA1/2 genes, including exons, some introns, and flanking splice site junctions. DNA was amplified using a Proflex thermocycler (Applied Biosystems) according to the manufacturer’s protocols. PCR products (200-800 bp) were analyzed using HT DNA extended range LabChip and the Caliber Lifescience analyzer and LabChip GX software (PerkinElmer,). Cleaning up of the PCR products was performed using ExoSAP-IT (GE Healthcare Life Sciences) following the manufacturer’s protocol. Sanger sequencing reactions were performed with BigDye Terminator v1.3 kits (Applied Biosystems) using forward and reverse sequencing and were analyzed on 3500 genetic analyzer (Applied Biosystems) with Sequence Pilot (GSI Medical Systems).

### Multiplex ligation-dependent probe amplification

Sequencing methods are limited in their capability to detect structural variants (SVs) due to short read utilization. Therefore, Multiplex ligation-dependent probe amplification (MLPA) was used in the analysis of BRCA1 and BRCA2 genes to ensure SVs are not undetected. For this, the SALSA MLPA Probemixes P002 BRCA1 and P090 BRCA2 (MRC Holland) were used; these contain 38 and 40 separate MLPA probes for BRCA1 and BRCA2, respectively. Fragments analyses were then carried out on the 3500 genetic analyzer (Applied Biosystems). The generated data were assessed using Coffalyser Net (MRC Holland) and interpretation was according to the manufacturer’s protocols.

### Variant interpretation

Data were analyzed using the Ion Reporter Software v5.2, which comprises a suite of bioinformatics tools for data analysis, annotation, and reporting of sequencing data from Ion Torrent instruments such as the ION S5 (Thermo Fisher Scientific). Classification of sequence variants was made as per the guidelines recommended by the American College of Medical Genetics and European Molecular Genetic Quality Network. Previously reported pathogenic mutations were considered pathogenic as inferred from the Universal Mutation Database (UMD, http://www.umd.be/BRCA1/ and http://www.umd.be/BRCA2/), Breast Cancer Information Core Database of National Institute of Health (BIC, http://research.nhgri.nih.gov/bic/) and literature evidence.

### Statistical analyses

Statistical analyses were performed using SPSS v29.0.1.0 to calculate mean and median, conduct chi-squared test for categorical variables correlation analysis, and Kaplan-Meier survival analysis. *P* = .05 or less was considered significant. Patients under age of 45 at time of diagnosis were considered as cases of EOBC, and at age of 45 or above were considered as LOBC.

## Results

### EOBC cases were frequent, and EOBC tumors showed more aggressive characteristics

A total of 1336 BC patients were included in the study; to the best of our knowledge, this is the largest published BC cohort from a MENA region. We defined 611 patients (45.7%) as EOBC cases (younger than 45 at diagnosis), and 725 (54.3%) as LOBC, confirming the high rate of EOBC in Oman. The median age of diagnosis with EOBC was 37 (range 22-44), while this was 50 for LOBC (range 45-95). Data concerning BC family history were available for 793 cases; 222 of these patients reported a family history (28%). Those with family histories were not significantly over-represented in the EOBC group (*P* = .398; Table 1), with a slightly smaller proportion of such cases (26.6%) compared to overall.

**Table 1.** EOBC cases were not more commonly associated with a BC family history than LOBC.

Characteristic	Age		*P* value
	Age < 45 (*n* = 387)	Age ≥ 45 (*n* = 406)	
Family history			0.398
Yes	103 (26.6%)	119 (29.3%)	
No	284 (73.4%)	287 (70.7%)	

Abbreviations: BC, breast cancer; EOBC, early-onset breast cancer; LOBC, late-onset breast cancer.

EOBC and LOBC cases were further compared in terms of standard clinicopathological prognostic factors (Table 2). EOBC tumors were significantly larger and of significantly higher grade than LOBC (*P* < .001), although they did not differ in terms of node positivity or expression of the proliferation marker Ki67. In addition, EOBCs were composed more of HER2-enriched and triple-negative breast cancer (ie, estrogen receptors negative, progesterone receptors negative, and HER2 receptors negative) subtypes than LOBCs, while LOBCs showed a higher percentage of luminal subtype than EOBCs (*P*.018).

**Table 2.** EOBC tumors were significantly larger and higher grade than LOBC tumors, and were enriched for aggressive subtypes.

Characteristic	Age		*P* value
	Age < 45 (*n* = 611)	Age ≥ 45 (*n* = 725)	
*Tumour size*			**<.001**
T0	17 (4.3%)	10 (2.0%)	
T1 (≤2 cm)	76 (19.0%)	152 (30.7%)	
T2 (>2 but ≤5 cm)	188 (47.0%)	229 (46.3%)	
T3 (>5 cm)	103 (25.8%)	76 (15.4%)	
T4	16 (4.0%)	28 (5.7%)	
*Nodal status*			.63
N0	144 (36.2%)	217 (44.8%)	
N1 (1-3)	120 (30.2%)	129 (26.7%)	
N2 (4-9)	80 (20.1%)	88 (18.2%)	
N3 (≥10)	54 (13.6%)	50 (10.3%)	
*Pathologic grade*			**<.001**
I	60 (11.6%)	130 (21.3%)	
II	277 (53.6%)	320 (52.5%)	
III	180 (34.8%)	160 (26.2%)	
*Ki-67*			.152
Mean	36.7	33.3	
Range	2-95	0-90	
*Subtype*			**.018**
Luminal	262 (44.9%)	380 (53.3%)	
Luminal-HER2	111 (19.0%)	116 (16.3%)	
HER2-enriched	109 (18.7%)	102 (14.3%)	
Triple negative	102 (17.5%)	115 (16.1%)	

Bold values represent significance with a *P* value of .05 or lower.

Abbreviations: EOBC, early-onset breast cancer; LOBC, late-onset breast cancer.

### BRCA1/2 gene mutations were not more common in EOBC

The primary aim of the study was to investigate whether EOBC cases in Oman were associated with increased prevalence of germline BRCA1/2 gene mutations. A total of 262 patients underwent germline genetic testing for BRCA1 and BRCA2 gene mutations; these cases comprised 187 EOBCs and 75 LOBCs. Overall, 41 patients tested positive for BRCA gene mutations, representing 15.6% of those tested. Thirty-two of these patients were within the EOBC group and 9 within the smaller LOBC group. Although the proportion of BRCA mutation carriers was numerically higher in EOBC as compared to LOBC (17.1% vs 12.0%) this was not significant (*P* = .229; Table 3).

**Table 3.** BRCA mutations were not more common in EOBC.

Characteristic	Age		*P* value
	EOBC (*n* = 187)	LOBC (*n* = 75)	
BRCA mutation status			.229
Positive	32 (17.1%)	9 (12.0%)	
Negative	155 (82.9%)	66 (88.0%)	

Abbreviation: EOBC, early-onset breast cancer; LOBC, late-onset breast cancer.

As a sense-check for our BRCA mutation data, we also assessed whether BRCA gene mutations were associated with reported BC family histories, as would be expected given that these mutations are the best-established inherited BC risk factor. Data were available for both BRCA mutations and family history in 180 cases (Table 4). BRCA genes mutations and BC family history were significantly positively associated (*P* = .017). In accordance with the high penetrance of these mutations, a large majority of patients with BRCA mutations reported BC family histories (72.2%), although other inherited factors are also strongly implicated since BRCA mutations were found in only a quarter of the total patients reporting family histories (26.5%).

**Table 4.** Family history of breast cancer and BRCA mutation status were positively associated.

Characteristic	BRCA mutation status		*P* value
	Negative (*n* = 144)	Positive (*n* = 36)	
Family history			**.017**
No	72 (50.0%)	10 (27.8%)	
Yes	72 (50.0%)	26 (72.2%)	

Bold values represent significance with a *P* value of .05 or lower.

Also, we compared BRCA mutation-positive and negative cancers in terms of standard clinico-pathological prognostic factors; we found no significant differences in tumor size, pathological grade, Ki67 scores, or molecular subtypes (Supplementary Table S1).

### Mutational characterization

The range of BRCA mutations within a large cohort of BC patients from Omani population has not previously been reported. Here, we report and characterize the mutations identified: 41 patients had a total of 42 mutations (Table 5). Overall, 12 mutations (28.5% of the total) were found in BRCA1, and 30 mutations (71.5%) were found in BRCA2. Of the 42 mutations identified, 26 were classified as pathogenic, while 16 were classified as variants of unknown significance (VUS). Mutations in EOBC were distributed as follows: 10 in BRCA1 (4 classified as pathogenic and 6 as variants of unknown significance, VUS), and 23 in BRCA2 (16 pathogenic and 7 VUS). For comparison, the 9 mutations identified in LOBC were distributed as follows: 2 in BRCA1 (1 pathogenic and 1 VUS) and 7 in BRCA2 (5 pathogenic and 2 VUS).

**Table 5.** Spectrum of BRCA gene mutations identified.

Age	Exon	Mutation	Protein	Type	Effect	Classification
	*BRCA1*					
EOBC	3	c.178C>T	p.Gln60Ter	Nonsense	Premature termination	Pathogenic
	2	c.68-69deIAG	p.Glu23fs	Deletion	Frameshift	
	10	c.895_896delGT	p.Val1299fs	Deletion	Frameshift	
	10	c.971G>T	p.Ser324Ile	Missense	Potential functional disruption	VUS
	6	c.398G>A	p.Arg133His	Nonsense	Potential deleterious	
	16	c.4993G>C	p.Val665Leu	Missense	Potential functional disruption	
	—	c.5452-6T>G^1^	p.[?]	Splice site	Potential functional disruption	
	22	c.5423T>C	p.Val1808Ala	Missense	Potential functional disruption	
LOBC	11	c.4165_4166delAG	p.Ser1389Ter	Deletion	Premature termination	Pathogenic
	10	c.2123C>A	p.Ser708Tyr	Nonsense	Potential deleterious	VUS
	*BRCA2*					
EOBC	10	c.1819A>T	p.Lys607Ter	Nonsense	Premature termination	Pathogenic
	3	Exon 3 deletion^3^		Deletion		
	11	c.5290_5291deITC	p.Ser1764fs	Deletion	Frameshift	
	11	c.5705delA^*^	p.Asp1902fs	Deletion	Premature termination	
	16	c.7673_7674delAG	p.Glu2558fs	Deletion	Frameshift	
	16	c.7679_7680de1TT	p.Phe2560fs	Deletion	Premature termination	
	23	c.9018C>A^2^	p.Tyr3006Ter	Nonsense	Premature termination	
	25	c.9382C>T^1^	p.Arg3128Ter	Nonsense	Premature termination	
	11	c.2588dupA^1^	p.Asn863fs	Nonsense	Frameshift	
	8	c.644_646delAAG	p.Glu215del	Deletion	Frameshift	VUS
	3	c.161dupA^*^	p.Asn54fs	Nonsense	Potential deleterious	
	10	c.1574C>G	p.Thr525Ser	Nonsense	Potential deleterious	
	10	c.1694C>T	p.Ala565Val	Missense	Moderate impact on protein structure	
	11	c.4045A>	p.lle1349Val	Missense	Likely benign	
	10	c.800G>A	p.Gly267Glu	Missense	Potential functional disruption	
	26	c.9586A>G	p.Lys3196Glu	Missense	Potential deleterious	
LOBC	11	c.2808_2811delACAA	p.Ala938fs	Deletion	Frameshift	Pathogenic
	11	c.3195_3198delTAAT	p.Asn1066fs	Deletion	Frameshift	
	11	c.4718delG^*^	p.Cys1573fs	Deletion	Frameshift	
	10	c.1423G>T	p.Glu475Ter	Nonsense	Premature termination	
	10	c.1794_1798delATCTT	p.Ser599Ter	Nonsense	Premature termination	
	20	c.8530G>A	p.Glu2844Lys	Nonsense	Potential deleterious	VUS
	—	c.8633-6T>A	p.[?]	Splice site	Potential functional disruption	

^1^Found in 2 unrelated patients.

^2^Found in 3 related patients.

^3^Found in 3 relative and 1 unrelated patients.

^*^Novel.

Abbreviation: VUS, variant of undetermined significance.

Four recurrent pathogenic mutations were detected in the BRCA2 gene. A whole exon 3 deletion was observed in 4 patients, 3 of whom were related. Additionally, C.9382C>T was found in 2 unrelated patients, C.9018C>A was identified in 3 related patients, and C.2588dupA was present in 2 unrelated patients. These 4 recurrent mutations collectively accounted for 36.6% (11 out of 30) of all the patients with mutant BRCA2. It is noteworthy that a single recurring intronic mutation (C.5452-6T>G) has been discovered in the BRCA1 gene among 2 unrelated patients residing in the southern region of Oman, specifically in the Dhofar governorate. Furthermore, our population has also presented 3 previously unidentified mutations in the BRCA2 gene: C.5705delA and C.4718delG, which are predicted to have pathogenic effects, and C.161dupA, which is classified as a VUS.

### Survival outcomes

**Figure 1.**
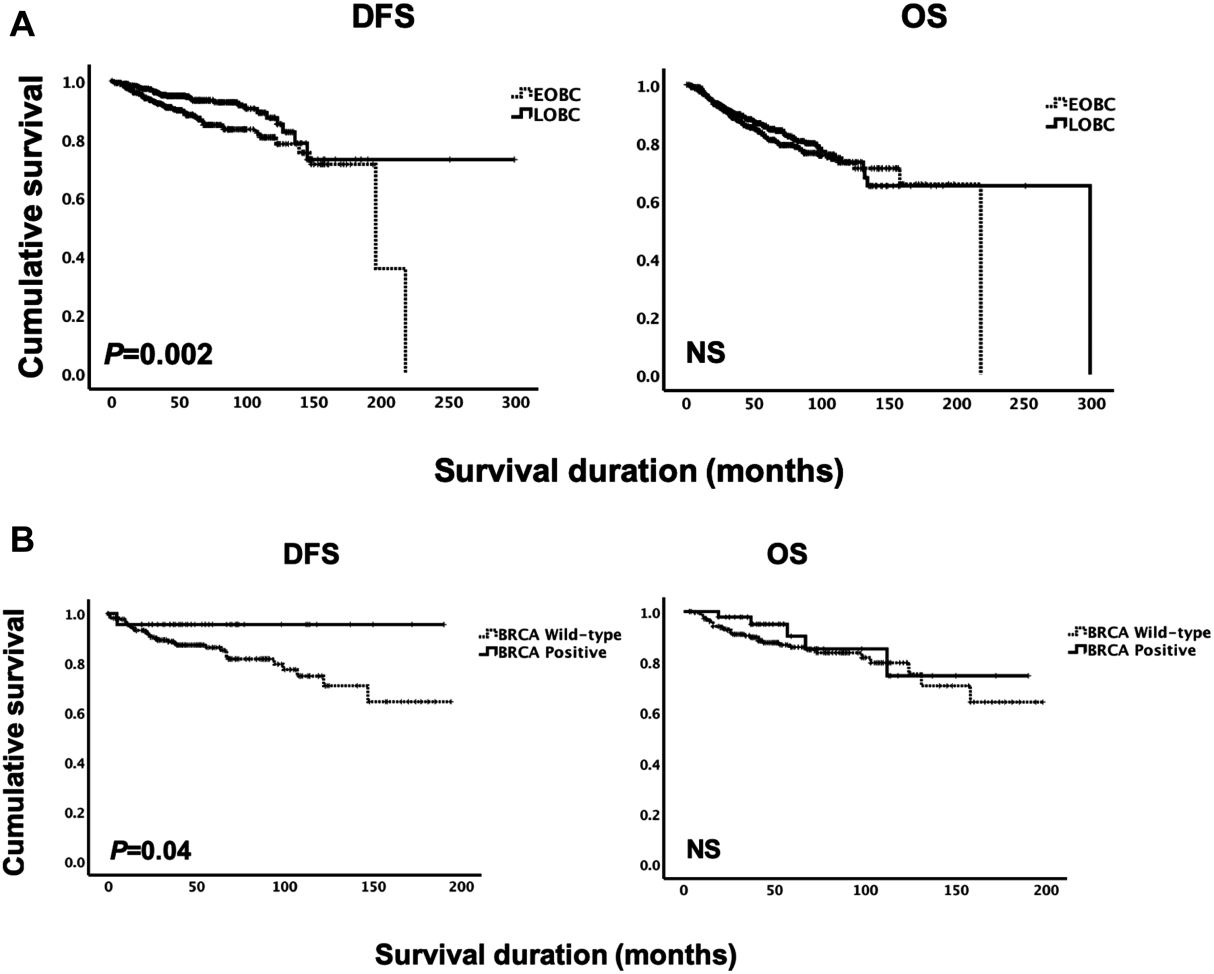
Kaplan-Meier survival analyses plots for DFS and OS. (A) DFS and OS curves for EOBC vs LOBC. (B) DFS and OS curves for BRCA-mutation positive vs BRCA wild-type. Abbreviation: DFS, disease-free survival; EOBC, early-onset breast cancer; LOBC, late-onset breast cancer NS, not significant; OS, overall survival.

Finally, we assessed the impacts of the age of onset (EOBC or LOBC) or BRCA mutational status (positive or wild-type) on cancer outcomes. The median follow-up period for this cohort was 49 months (range: 2-300 months). Kaplan-Meier survival analyses were used to compare outcomes between EOBC and LOBC in terms of both disease free (DFS) and overall survival (OS) (Figure 1A). EOBC patients had a significantly shorter DFS than LOBC by a mean of 76 months (*P* = .002; EOBC 170 months [95% IC, 155-185] vs LOBC 246 months [95% CI, 221-270]. EOBC patients also had numerically shorter OS by 54 months, although this was not significant (*P* = .38; EOBC 167 months [95% CI 156-178] vs LOBC 221 months [95% IC 201-240].

Further analyses were performed to determine survival outcomes among BRCA-positive versus BRCA wild-type cases (Fig 1B). BRCA-positive cases showed significantly better DFS than BRCA wild-type by 12 months (*P* = .04; positive 182 months [95% CI, 171-193] vs wild-type 151 months [95% CI, 138-165]), while—surprisingly—this was not significant in terms of overall survival (*P* = .44).

## Discussion

To the best of our knowledge, this is the largest study in which BC patients from the Middle East and North Africa (MENA) region have been characterized at the clinico-pathological and genetic levels. The primary aim of the study was to investigate the reported high incidence of EOBC in Oman, and to examine to what extent these cases were associated with BRCA1/2 gene mutations, which are well known to pre-dispose to EOBC. For example, BRCA1/2 mutations have been found to be approximately 6-fold enriched in EOBC populations as compared to later onset cohorts.^12^

Firstly, we confirmed the very high proportion of early onset cases within our overall BC cohort at 45.7%. This rate is more than 5-fold higher than typical figures for Western populations^7^ and is higher than any figure we have found reported in the literature. Next, we determined that these EOBC cases were not significantly associated with a greater proportion of BRCA1/2 germline mutations than in LOBC cases. Our data do not give any insight into the prevalence of BRCA1/2 mutations in the Omani population overall, since we offered genetic testing only to those with BC and with a high likelihood of an inherited BC pre-disposition, but they do strongly suggest that the high rate of EOBC is not driven in the main by BRCA1/2 mutations. This differs from findings in a Korean-population,^16^ a Japanese-population,^17^ and various other western populations based studies,^12,18,19^ and indicates other causative factors that are specific to Oman. Furthermore, it should be noted that epigenetic deregulation has been reported to damage the function of BRCA1, via increases in promotor DNA methylation, changes to histone modifications.^20-22^ The epigenetic alterations in BRCA1/2 genes could possibly have contributed to our findings, although we have not investigated this further.

This unexpected finding prompted us to investigate whether EOBC cases in Oman were associated with family history of BC, which again showed no significant correlation; the majority of EOBC cases (73.4%) reported no family history suggesting non-hereditary factors may have a role in Oman. This finding appears to contradict the global findings in which EOBC cases were more likely to report at least 1 first-degree relative diagnosed with BC.^18,19,23^ However, our results agree with data from international studies showing a significant correlation between BRCA gene mutations and family history.^24,25^

EOBC cancers were next compared in terms of clinicopathological features to LOBCs, which revealed that EOBCs were significantly larger, of higher grade, and of more aggressive subtypes. These findings are broadly consistent with studies performed in a population from the neighboring country of Saudi Arabia^26^ and from other populations in Africa, including Ghana, Tanzania, and Tunisia, which show EOBC to be associated with a variety of different poor prognostic factors with some variation in whether significant findings were found for size, grade, Ki-67 expression levels, or lymph nodal status.^27-29^ We also found EOBC to have significantly shorter DFS, which can potentially be explained in part by the poor prognostic features. Similar findings have been reported in various populations,^30^ some of which have had the statistical power to demonstrate in multi-variate analyses that EOBC itself was an predictor of poor prognosis independently of standard prognostic factors,^31,32^ hinting at aggressive biology that differs in more subtle ways.^7^ However, this remains controversial with some reports showing no significant difference in outcomes in EOBC^31^ and others suggesting the difference only exists in the better prognosis subtypes.^33^

Tumors in BRCA mutation carriers did not significantly differ in clinicopathological features from those in BRCA wild-type patients in our data, but despite this, they were associated with better DFS. This differs from the literature in a number of regards. Firstly, breast tumors in BRCA mutation carriers have long been reported to show poorer prognostic features than wild-type patients; for example, BRCA1 mutant tumors tend to be a higher grade and are more frequently triple-negative, although this is less marked and less consistently observed in the literature for BRCA2.^34,35^ Our study may be limited in terms of patient numbers, and in particular in mutations in BRCA1 that are reported to show the stronger differences, to draw a confident conclusion. With respect to survival, the literature is conflicting to some extent, for example, studies have been published that show no difference in survival between BRCA mutation carriers vs BRCA wild-type,^36^ others contradict our findings,^37^ while others agree.^38^ It is likely that these differences relate to variations in case-mix, mutations (specifically proportions of BRCA1 or BRCA2), and in treatments offered. Recent reports show subgroups within BRCA mutation carriers to have significant differences from each other and relative to wild-type; for example BRCA mutant triple negative cancer, but not luminal cancers, have improved outcomes compared to wild-type^39^ and similar differential responses are seen with platinum-based therapies and—most recently—with PARP inhibitors.^40^

In terms of mutations identified, the distribution showed BRCA2 mutations were the majority (71.5%), which is in alignment with other reports in Asian populations but not in Western populations,^41,42^ highlighting that ethnic differences are prevalent and that studies into specific populations are essential to guide treatment in these regions. We also uncovered a distinct range of mutations in BRCA2, including 4 recurrent pathogenic mutations (exon 3 deletion, C.9382C>T, C.9018C>A, and C.2588dupA) that collectively accounted for 37% of patients with mutant BRCA2. Interestingly, none of these mutations were reported previously in Arab regions, and were only rarely reported in some other Western countries.^43-46^ This indicates the Omani population possesses a BRCA mutational spectrum that is different from the other MENA populations. Also, these mutations potentially represent founder mutations in the Omani population, which could be used in targeted screening for BRCA2 gene mutations as a cost- and time-effective alternative to comprehensive gene screening. However, a large-scale cohort study is required to validate the applicability and founder mutation status of these mutations from unselected patients, particularly as at this stage we found the C.9018C>A mutation only within members of one family. Also, genotype and haplotype analyses are required to confirm whether these mutations relate to a common ancestor and therefore identified as founder mutations. Furthermore, a recurrent VUS was found in 6 nucleotides upstream of the splice site between intron 20 and exon 20 in BRCA1. A previous publication reported this mutation as being pathogenic solely on the basis of mutation frequency and without experimental evidence.^47^ Interestingly, we discovered this mutation in the BRCA1 gene in 2 unrelated patients residing in the southern region of Oman, specifically in the Dhofar governorate. This mutation may prompt further validation to assess its pathogenicity in BC and may serve as a founder mutation specifically for this part of the country. Additionally, we have discovered 3 mutations that have not been previously reported in the Breast Information Core (BIC) database, or Universal Mutation Database (UMD); they are unique to our population (namely C.5705delA, C.4718delG, and C.161dupA). In other Arab regions, some other studies have also reported novel mutations in BRCA genes in Arab regions, indicating there is a distinct mutational spectrum in this part of the world, supporting the need for genetic databases for the MENA region.^48,49^ Reporting of these variants is important to allow future assessments of their potential pathogenic roles^50^

## Conclusion

We have demonstrated that EOBC is unusually common in Oman, but is not associated with germline BRCA1/2 gene mutations. Our recommendation is to expand testing of young BC patients in the region for autosomal dominant gene mutations that are associated with hereditary BC including ATM, CHEK2, PALP2, PTEN, and TP53. Also, consideration should be given to sequencing tumor cells for somatic mutations in these genes that are associated with EOBC.

## Supplementary material

Supplementary material is available at *The Oncologist* online.

oyae214_suppl_Supplementary_Tables_S1_Figures_S5

## Data Availability

All data are available either within the manuscript and supplementary materials, or directly from the corresponding author.
