# Supplementary material for: BRCA1/2 mutations and outcomes among Middle Eastern patients with early-onset breast cancer in Oman
Source: Oncologist. 2024 Aug 26;29(12):e1714–22. doi: 10.1093/oncolo/oyae214 (PMC11630774; doi:10.1093/oncolo/oyae214)
Supplement: oyae214_suppl_Supplementary_Tables_S1_Figures_S5 [file oyae214_suppl_supplementary_tables_s1_figures_s5.docx]

| **Characteristic** | **Age** | | **P Value** |
| --- | --- | --- | --- |
|  | BRCA-wildtype (n=221) | BRCA-positive (n=41) |  |
| **Tumour size** |  | | <0.656 |
| T0 | 9 (5.7%) | 1 (2.9%) |  |
| T1 (≤ 2cm) | 40 (25.5%) | 6 (17.1%) |  |
| T2 (> 2 but ≤ 5cm) | 69 (43.9%) | 20 (57.1%) |  |
| T3 (> 5cm) | 35 (22.3%) | 7 (20.0%) |  |
| T4 | 4 (2.5%) | 1 (2.9%) |  |
| **Nodal Status** |  | | <0.380 |
| N0 | 71 (45.2%) | 10 (29.4%) |  |
| N1 (1-3) | 43 (27.4%) | 13 (38.2%) |  |
| N2 (4-9) | 29 (18.5%) | 7 (20.6%) |  |
| N3 (≥ 10) | 14 (8.9%) | 4 (11.8%) |  |
| **Pathologic Grade** |  | | <0.739 |
| I | 19 (11.4%) | 3 (7.7%) |  |
| II | 89 (53.6%) | 23 (59.0%) |  |
| III | 58 (34.9%) | 13 (33.3%) |  |
| **Ki-67** |  | | <0.200 |
| Mean | 38 | 40 |  |
| Range | 1-90 | 15-80 |  |
| **Subtype** |  | | <0.499 |
| Luminal | 89 (43.2%) | 22 (48.9%) |  |
| Luminal-HER2 | 33 (16.0%) | 8 (17.8%) |  |
| HER2-enriched | 25 (12.1%) | 2 (4.4%) |  |
| Triple Negative | 59 (28.6%) | 13 (28.9%) |  |
